# Diagnostic Utility of Automated Breast Volume Scan in Differentiating Benign and Malignant Breast Masses

**DOI:** 10.7759/cureus.87191

**Published:** 2025-07-02

**Authors:** Ranjini Rajkumar, Tulika Singh, Veenu Singla, Gurpreet Singh, Amanjit Bal, N Khandelwal

**Affiliations:** 1 Department of Radiodiagnosis and Imaging, Postgraduate Institute of Medical Education and Research Chandigarh, Chandigarh, IND; 2 Department of General Surgery, Postgraduate Institute of Medical Education and Research Chandigarh, Chandigarh, IND; 3 Department of Pathology, Postgraduate Institute of Medical Education and Research Chandigarh, Chandigarh, IND

**Keywords:** automated breast volume scan, birads, breast cancer, breast ultrasound, retraction phenomenon

## Abstract

This study aims to compare the diagnostic value of Automated Breast Volume Scan (ABVS) and Hand-Held Ultrasound (HHUS) in the characterization of breast masses and in differentiating benign and malignant breast masses. This is a prospective study on 101 patients with breast masses referred for ultrasound-guided biopsy. All patients underwent both HHUS (routine breast ultrasound) and ABVS of bilateral breasts and were classified according to the Breast Imaging-Reporting and Data System (BI-RADS). All ultrasound parameters to assess the lesions were compared for sensitivity, specificity, and accuracy between the two modalities. All the patients enrolled underwent ultrasound-guided biopsy of the breast lesions.

One hundred eight breast lesions were identified and characterized in the 101 patients studied. The negative predictive value (NPV) of BIRADS-3 lesions in excluding malignancy was 82.6% and 90% by HHUS and ABVS, respectively. The positive predictive value (PPV) of BIRADS-5 lesions in predicting malignancy was 94.7% and 98.4% by HHUS and ABVS, respectively. The two ultrasound (US) modalities showed substantial agreement (k = 0.613). The most sensitive lesion characteristic suggesting malignancy was found to be irregular shape on both ABVS and HHUS. The retraction phenomenon identified in the coronal plane of ABVS was 100% specific and had a PPV of 100% in predicting a malignant lesion. As the diagnostic accuracy is similar in both modalities for breast masses, ABVS proves to be a promising diagnostic tool for breast lesions with the advantages of being reproducible, operator-independent, and standardized.

## Introduction

Breast cancer remains the most common malignancy among women worldwide. In 2022, nearly 2.3 million new cases were diagnosed globally, accounting for approximately 670,000 cancer-related deaths [1]. Regular screening and early detection have been shown to significantly reduce breast cancer mortality [2]. The primary goal of screening is to detect malignancies at an asymptomatic and early stage, allowing for less invasive treatment options and improved patient outcomes [3].

Mammography has long been regarded as the gold standard for breast cancer screening [4-6]. However, it is associated with certain limitations. Mammography exposes patients to ionizing radiation, has a relatively high false-positive rate, and may contribute to overdiagnosis, all of which can elevate patient anxiety [7,8]. Furthermore, its sensitivity is reduced in women with dense breast tissue, who are also at an increased risk of developing breast cancer [9,10]. This limitation is particularly significant in women under 50 years of age, potentially reducing the effectiveness of screening programs in this population [11].

Ultrasound serves as a valuable adjunct to mammography, especially in women with dense breasts [12,13]. However, conventional hand-held ultrasound (HHUS) is highly operator-dependent, requiring real-time diagnosis during image acquisition or review of a limited number of static images. This introduces variability in interpretation, increases recall rates, prolongs examination time, and contributes to patient anxiety [14,15]. Consequently, HHUS is not routinely utilized in population-based screening programs, though it is employed selectively in cases where mammography is less sensitive.

To address the limitations of HHUS, an operator-independent modality, the Automated Breast Volume Scanner (ABVS), was developed. ABVS enables the acquisition of a complete series of B-mode images covering the entire breast volume, which are reconstructed in three orthogonal planes. These images can be reviewed independently by a radiologist at a dedicated workstation. ABVS offers a standardised, reproducible approach to breast imaging and has been shown to reduce patient recall rates compared to HHUS [16]. The U.S. Food and Drug Administration (FDA) approved ABVS in 2012, recognizing its efficacy in detecting small lesions in dense breast tissue [17]. The present study aims to compare HHUS and ABVS in the characterization of breast lesions. Specifically, we assess the efficacy of ABVS in differentiating benign from malignant lesions and evaluate the respective advantages and disadvantages of each modality. All imaging findings were correlated with histopathological outcomes.

## Materials and methods

A prospective comparative study between ABVS and HHUS was done at our institute after being approved by the Institutional Ethics Committee, Postgraduate Institute of Medical Education and Research Chandigarh with approval number NK/1616/MD/10519-20. The initial evaluation of the patients in our study was done by an experienced surgeon at the breast clinic. Women with palpable lumps were referred to radiology department for mammography or ultrasound. 

**Figure 1:**
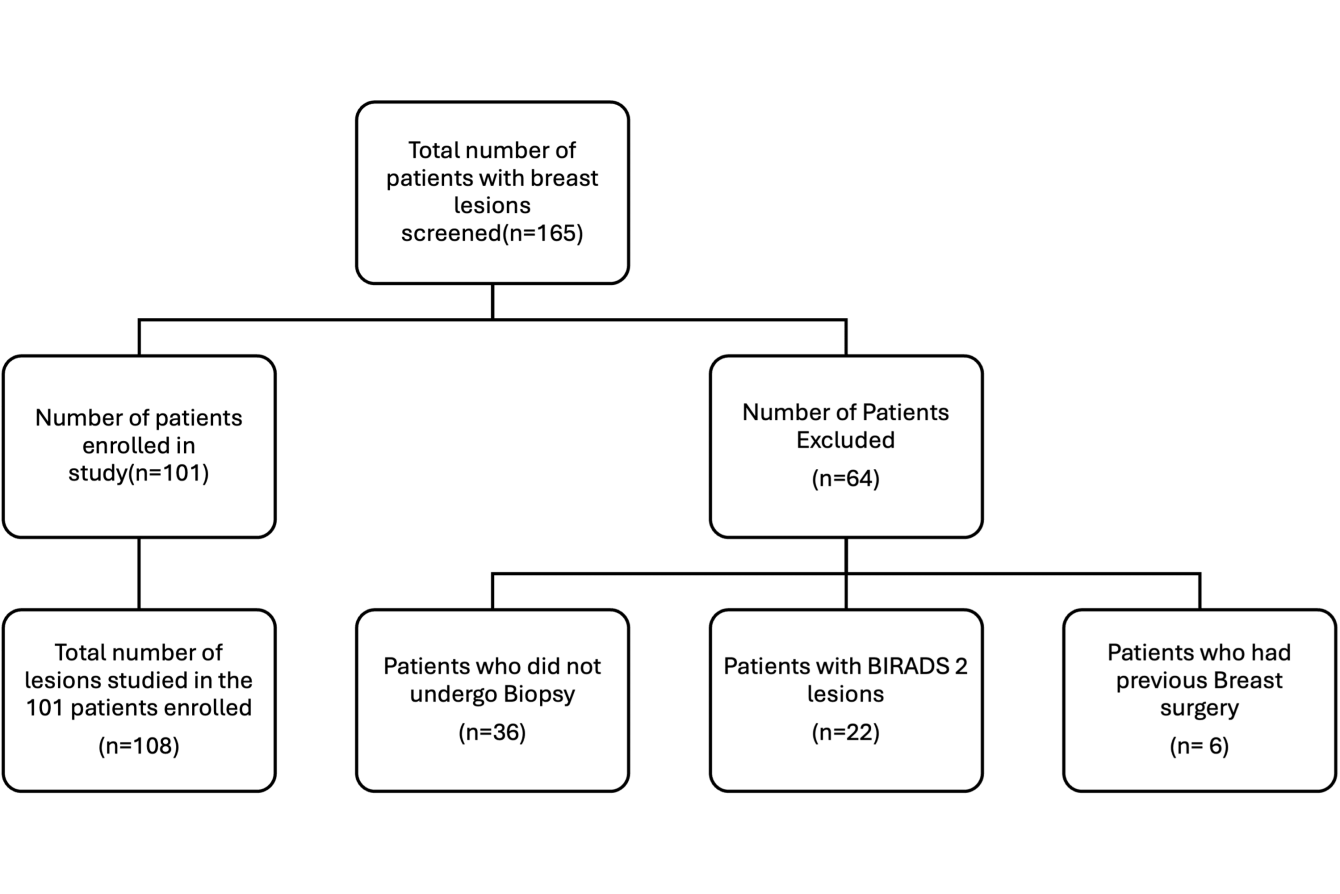
Flow diagram of study showing patients screened, excluded and enrolled.

A total of 165 consecutive patients with breast lesions were evaluated by mammography or ultrasound. The inclusion criteria for our study were women who presented with lesions classified as BIRADS 3,4 and 5 on mammography or ultrasound. Patients with BIRADS 2 lesions were excluded as biopsy was not warranted in these lesions. Patients with painful breast condition/ulcers who were not amenable for compression and those who underwent any kind of breast surgery in the past were excluded. Of the 165 patients evaluated, 64 patients with BIRADS 2 lesions, previous breast surgeries, and those not willing to undergo a biopsy were excluded.Below is a patient recruitment flow diagram (Figure 1).

A total of 101 patients (202 breasts) were enrolled in the study. Informed consent was obtained from each patient. These patients had ABVS examination and hand held ultrasound done. All the patients enrolled underwent ultrasound guided biopsy of the breast lesions.

Automated breast volume scan and ultrasound equipment

ABVS was performed using the ACUSON S2000 automated breast volume scan (ABVS; Siemens Medical Solutions, Mountain View, CA, USA) consisting of the ABVS module with the core components of flexible arm, touch screen monitor, and scanner (transducer that employs frequencies of 5-14 MHz, scan box and screen membrane for contact) and a 3D workstation. ABVS of both breasts were done in three planes, (medial, lateral and anteroposterior (AP) view) which took approximately 20 minutes to acquire. Additional views were taken if a lesion was in the periphery and could not be covered in the routine views and in patients with large breasts. ABVS interpretation was done by two radiologists who had more than 10 years of experience in breast imaging and had two years experience in reporting ABVS. The examiners were blinded to the results of HHUS. HHUS was done on ACUSON S2000 using linear array transducer with 5-12 MHz frequency by a third radiologist who was blinded to the results of ABVS.

Study description

Ultrasound features of the lesions were recorded in a database which included antero-posterior width ratio, shape, margin, lesion echogenicity, and posterior acoustic phenomena. In addition retraction phenomenon if identified was noted in ABVS. With these features the lesions were given a BIRADS score according to American College of Radiology (ACR) BIRADS lexicon for breast ultrasound, by the sonologist. The same parameters were used to classify lesions by ABVS. The final outcome was compared with the histopathological results.

Statistical analysis

All Ultrasound parameters to characterise the lesions were compared between both the modalities along with the gold standard histopathological results. Logistic regression was applied to each parameter. The sensitivity, specificity, PPV and NPV of individual parameter in predicting malignancy was calculated. Qualitative and categorical variables were described as frequencies or proportions and compared using Chi squared test for degree of association. McNemars test for significance of changes between the two modalities was applied. The degree of agreement between the two modalities and inter observer reliability was studied using kappa statistics. Landis and Koch kappa scale was used to interpret the kappa statistics with 0-0.2, 0.2-0.4, 0.4-0.6, 0.6-0.8, 0.8-1 representing slight, fair, moderate, substantial and almost perfect agreement respectively. The significance of difference between the sensitivity and specificity of ABVS and HHUS was tested using McNemar Chi-square test. All statistical tests were two sided and were performed at a significance level of α=0.05.

## Results

A total of 101 patients presenting with breast lesions were included in the study. A total of 108 breast lesions were identified and assessed. Each lesion was evaluated using both hand-held ultrasound (HHUS) and automated breast volume scanning (ABVS), with findings compared between the two modalities and validated against histopathological outcomes.

Histopathological classification of lesions

Of the 108 breast lesions studied, histopathological examination revealed that 72 66.7%) lesions were malignant and 36 (33.3%) were benign. The distribution of histopathological diagnoses is shown in Table 1.

**Table 1:** Histopathological classification of breast lesions

Type	Pathological types of lesions	Number of patients (N), %
Benign	Fibroadenoma	18 (16.6%)
	Fibroepithelial lesion	4 (3%)
	Phyllodes tumour	3 (2%)
	Fat necrosis	1 (0.9%)
	Tubular adenoma	1 (0.9%)
	Fibrocystic disease	1 (0.9%)
	Benign breast tissue/no e/o malignancy	3 (2.7%)
	Intraductal papilloma	2 (1.8%)
	Graulomatous mastitis	1 (0.9%)
	Sclerosing adenosis	1 (0.9%)
	Benign spindle cell tumour	1 (0.9%)
	Total	36 (33.3%)
Malignant	Infiltrating ductal carcinoma	66 (61.1%)
	Ductal carcinoma in situ	1 (0.9%)
	Medullary carcinoma	1 (0.9%)
	Micropapillary invasive carcinoma	1 (0.9%)
	Infiltrating lobular carcinoma	2 (1.8%)
	Solid papillary carcinoma with infiltration	1 (0.9%)
	Total	72 (66.7%)

Most lesions (66.7%) in this study were histologically malignant. This contrasts with prior studies in which benign lesions were more commonly analyzed for sonographic characterization using ABVS and HHUS [18,19]. The higher malignant yield in our study may be attributed to our inclusion criteria, which limited analysis to BIRADS 3-5 lesions, whereas other studies have included BIRADS 1-5 [20].

Lesion characteristics

**Figure 2:**
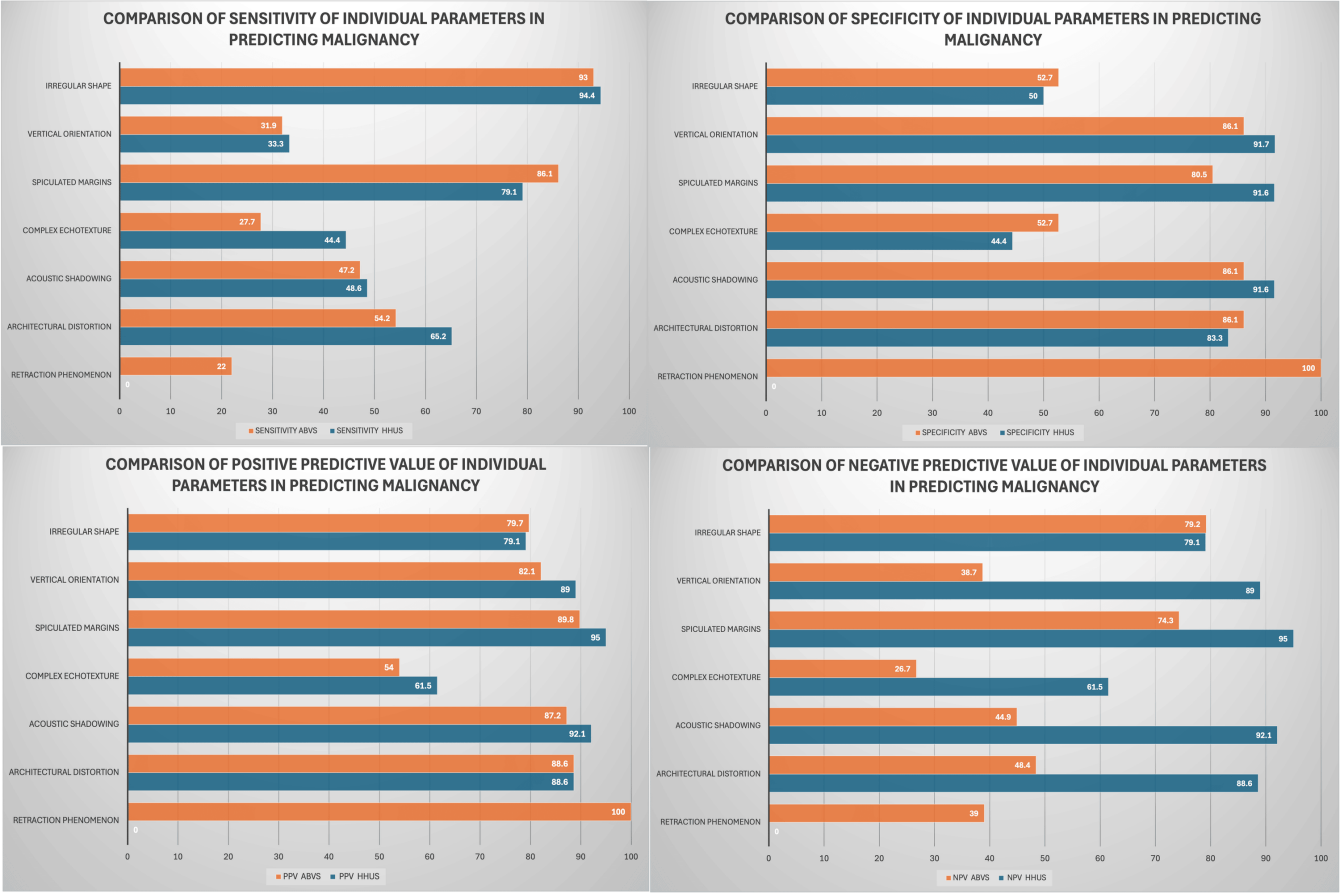
Malignancy prediction parameters Graphs showing sensitivity, specificity, positive predictive values, and negative predictive values of lesion characteristics in predicting malignancy.

Lesion characteristics evaluated included shape, orientation, margin, echogenicity, acoustic features, calcification, cystic changes, and architectural distortion. These features were analyzed using both HHUS and ABVS and compared against the histopathological gold standard. Sensitivity, specificity, positive predictive value (PPV), and negative predictive value (NPV) for each feature in predicting malignancy are depicted in Figure 2.

Using HHUS, the most sensitive feature for predicting malignancy was an irregular shape (94%), while the most specific features were the lesion margins, vertical orientation, and acoustic shadowing (each at 92%). For ABVS, the most sensitive feature was also irregular shape (93%), while the most specific were vertical orientation, architectural distortion, and acoustic shadowing (each at 86%). Notably, the retraction phenomenon in the coronal plane seen on ABVS demonstrated 100% specificity and PPV for malignancy.

BIRADS categories of the lesions

Based on the ACR BIRADS lexicon, HHUS classified 23 lesions (21%) as BIRADS 3, 28 (26%) as BIRADS 4, and 57 (53%) as BIRADS 5. ABVS classified 20 lesions (19%) as BIRADS 3, 27 (25%) as BIRADS 4, and 61 (56%) as BIRADS 5. ABVS demonstrated higher sensitivity (83%) than HHUS (75%) in detecting malignancies, with the difference being statistically significant (p = 0.038). ABVS also showed higher specificity (97.2%) compared to HHUS (91.6%), although this was not statistically significant (p = 0.067). The positive predictive value (PPV) and negative predictive value (NPV) of BIRADS scores 3, 4, 5 in detecting malignant lesions by both HHUS and ABVS are represented in Table 2.

**Table 2:** PPV and NPV of individual BI-RADS categories by ABVS and HHUS PPV: Positive predictive value, NPV: Negative predictive value, BI-RADS: Breast imaging-reporting and data system, ABVS: Automated breast volume scan, HHUS: Hand-held ultrasound.

BIRADS (ABVS)	PPV	NPV	BIRADS ( HHUS)	PPV	NPV
3	10% (2/20)	90% (18/20)	3	17.4% (4/23)	82.6% (19/23)
4	37% (10/27)	63% (17/27)	4	50% (14/28)	50% (14/28)
5	98.4% (60/61)	1.6% (1/61)	5	94.7% (54/57)	5.3% (3/57)

Comparison of lesion characteristics between HHUS and ABVS

Kappa statistics were used to evaluate agreement between ABVS and HHUS for lesion characterisation. The agreement between HHUS and ABVS in categorizing each lesion characteristic is given by a series of kappa coefficient values as in Table 3.

**Table 3:** Agreement of individual lesion characteristics between ABVS and HHUS Three of the eight lesion characteristics (shape, margin, cystic changes) showed substantial agreement between the two modalities, while five others demonstrated moderate agreement. Agreement on BIRADS categorisation was also substantial (κ = 0.613, p < 0.001), confirming consistency between hand-held ultrasound (HHUS) and automated breast volume scan (ABVS). The p-value of all the observations were significant (p<0.001), indicating that the kappa coefficient of agreement for various lesion characteristics did not occur by chance.

Lesion characteristics	Kappa coefficient	p Value	Agreement by Landis and Koch
Shape	0.724	<0.001	Substantial
Orientation	0.537	<0.001	Moderate
Margin	0.758	<0.001	Substantial
Echogenicity	0.306	<0.001	Fair
Cystic changes	0.62	<0.001	Substantial
Calcifications	0.57	<0.001	Moderate
Acoustic features	0.502	<0.001	Moderate
Architectural distortion	0.573	<0.001	Moderate
BIRADS score	0.613	<0.001	Substantial

A cross-tabulation comparing BIRADS by ABVS versus HHUS is given in Table 4. 

**Table 4:** Cross tabulation of BI-RADS according to ABVS and HHUS BI-RADS: Breast imaging-reporting and data system, ABVS: Automated breast volume scan, HHUS: Hand-held ultrasound.

	BI-RADS BY HHUS				
BI-RADS BY ABVS	BI-RADS SCORE	3	4	5	TOTAL
	3	14 (70%)	5(25%)	1(5%)	20 (19%)
	4	6 (22%)	16 (59%)	5 (19%)	27 (25%)
	5	3 (5%)	7 (11%)	51 (84%)	61 (56%)
	TOTAL	23 (21%)	28 (26%)	57 (53%)	108

Inter-observer reliability of ABVS

To evaluate inter-observer agreement, two independent radiologists assessed the ABVS data. The agreement for each lesion characteristic is shown in Table 5.

**Table 5:** Inter-observer agreement of ABVS interpretation Landis and Koch's kappa scale was used to interpret the kappa statistics with 0-0.2, 0.2-0.4, 0.4-0.6, 0.6-0.8, 0.8-1 representing slight, fair, moderate, substantial, and almost perfect agreement, respectively. Inter-observer agreement between the two independent observers was almost perfect in characterising the lesions. ABVS: Automated breast volume scan.

Lesion characteristics	Kappa coefficient	Agreement by Landis and Koch
Shape	0.952	Almost perfect agreement
Orientation	0.841	Almost perfect agreement
Margin	0.945	Almost perfect agreement
Echopattern	0.715	Substantial agreement
Posterior acoustic features	0.827	Almost perfect agreement
Calcifications	0.896	Almost perfect agreement
Architectural distortion	0.846	Almost perfect agreement
Hyperechoic rim	0.884	Almost perfect agreement
Retraction phenomenon	0.863	Almost perfect agreement
BI-RADS	0.828	Almost perfect agreement

Interpretation based on Landis and Koch's scale confirms that inter-observer reliability for ABVS was almost perfect in most lesion characteristics, underscoring the reproducibility of this modality.

Detection of additional lesions by ABVS

All lesions detected by HHUS were also identified on ABVS. Additionally, ABVS identified 21 lesions not seen on HHUS. Of these, nine lesions (42%) were BI-RADS 2 (mostly simple cysts), two lesions (9%) were BI-RADS 3, five lesions (23%) were BI-RADS 4, five lesions (23%) were BI-RADS 5. Notably, 10 lesions categorised as BIRADS 4 or 5, which were either suspicious or highly suggestive of malignancy, were missed by HHUS but detected by ABVS.

## Discussion

Our study demonstrates that automated breast volume scanning (ABVS) is a reliable and diagnostically valuable modality for evaluating breast lesions, showing comparable, if not superior, performance to handheld ultrasound (HHUS). Among 108 lesions in 101 patients, ABVS identified all lesions detected by HHUS and revealed 21 additional lesions, including 10 classified as BI-RADS 4 or 5, six of which were confirmed as multifocal or multicentric malignancies. This finding has significant implications for surgical planning and treatment stratification.

Diagnostic accuracy and BI-RADS categorisation

Our findings demonstrate that ABVS shows higher diagnostic accuracy than HHUS. For BI-RADS 5 lesions, ABVS demonstrated higher sensitivity (83.3%) and specificity (97.2%) compared to HHUS (75% and 91.6%, respectively). Similarly, BI-RADS 3 lesions had a higher negative predictive value (NPV) with ABVS (90%) versus HHUS (82.6%). These results are consistent with previous studies, such as those by Kotsianos-Hermle et al. [20] and Wang et al. [21], which reported ABVS as a reliable modality with high sensitivity and specificity (Table 6). Such performance metrics underscore ABVS’s potential to enhance diagnostic confidence, particularly in malignancy risk stratification.

**Table 6:** Comparison of sensitivity and specificity of BI-RADS categories by HHUS and ABVS between various studies and the present study BI-RADS: Breast imaging-reporting and data system, ABVS: Automated breast volume scan, HHUS: Hand-held ultrasound.

Study	Number of patients	Sensitivity		Specificity	
		HHUS	ABVS	HHUS	ABVS
Wang et al. (2012) (21)	175	90.60%	95.30%	82.50%	80.50%
Chen et al. (2013) (18)	213	88%	92.50%	87.50%	86.20%
Kotsianos-Hermle et al. (2009) (20)	97	97.50%	96.50%	88.50%	92.30%
Present study (BI-RADS 4 & 5 combined)	101	94.40%	97.20%	53%	50%
Present study (BI-RADS 5 lesions alone)	101	75%	83%	91.60%	97.20%

Lesion characteristics predictive of malignancy

**Figure 3:**
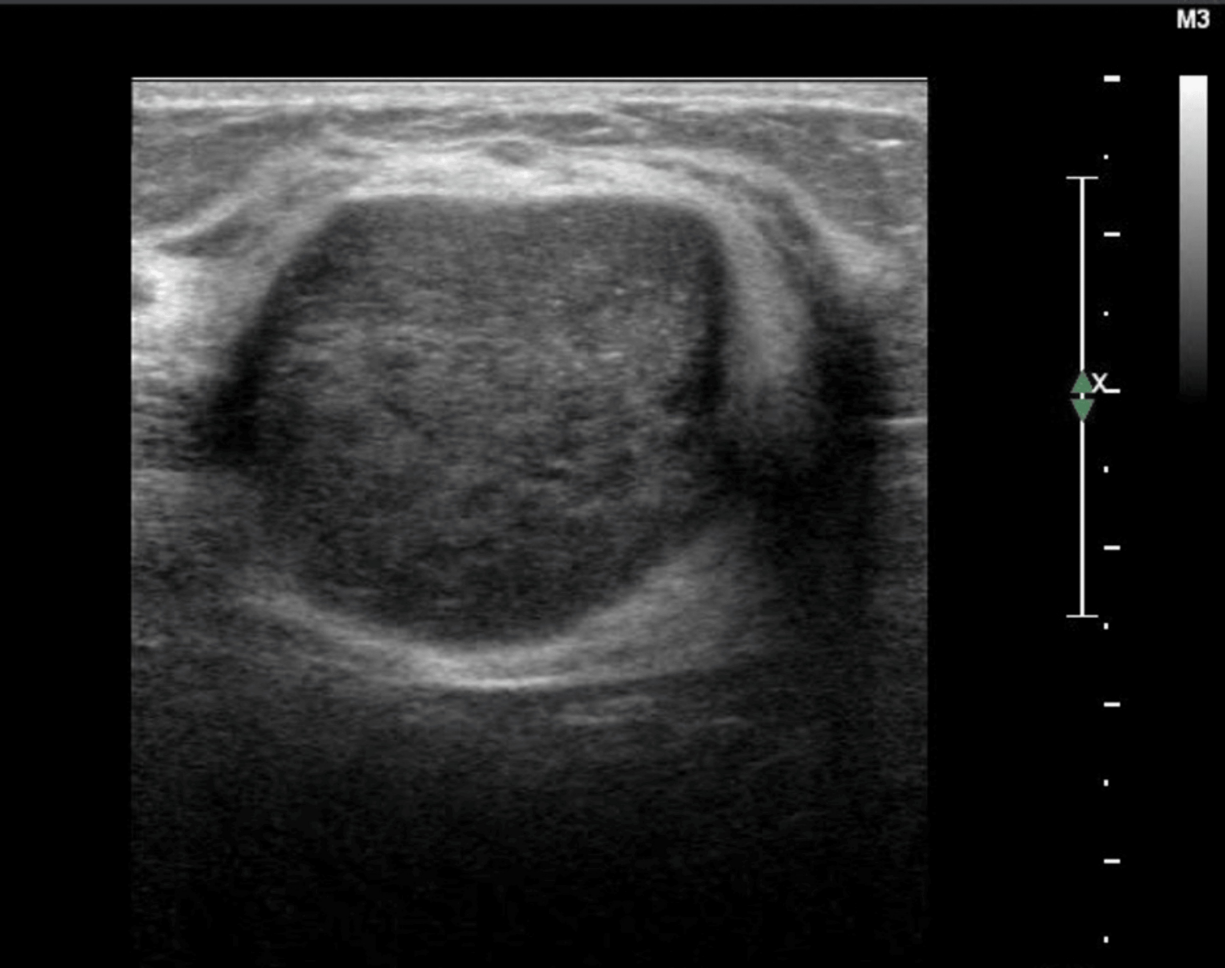
HHUS image of a 30-year-old patient The hand-held ultrasound. (HHUS) image is showing well-circumscribed hypoechoic lesion with a smooth margin at the 12 o'clock position of the left breast.

**Figure 4:**
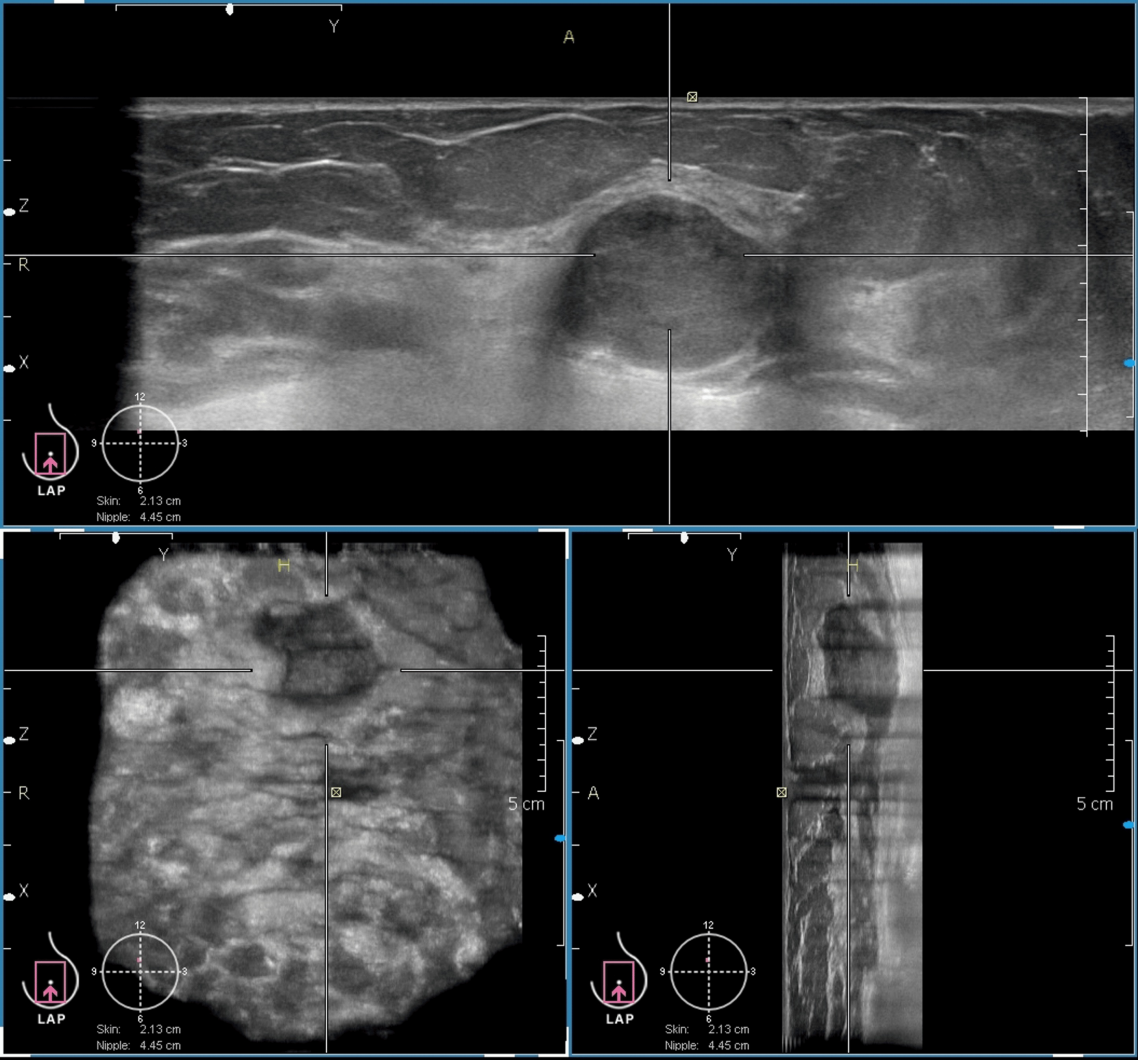
ABVS image of the 30-year-old patient The image is demonstrating a lesion at the 12 o'clock position of the left breast, consistent with a fibroadenoma. The lesion exhibits imaging characteristics similar to those observed on HHUS, highlighting the concordance between both modalities in lesion assessment. ABVS: Automated breast volume scan, HHUS: Hand-held ultrasound.

We found that several sonographic features-irregular shape, vertical orientation, indistinct or spiculated margins, posterior acoustic shadowing, architectural distortion, and higher BI-RADS categories-were significantly associated with malignancy on both ABVS and HHUS. Among these, “margin” characteristics stood out as the most predictive, consistent with earlier findings by Kotsianos-Hermle et al. [20]. Notably, spiculated margins had a 100% positive predictive value across both imaging modalities. This finding reinforces the importance of detailed margin analysis in differentiating benign from malignant lesions. Figures 3, 4 show a case of fibroadenoma demonstrating smooth margins.

**Figure 5:**
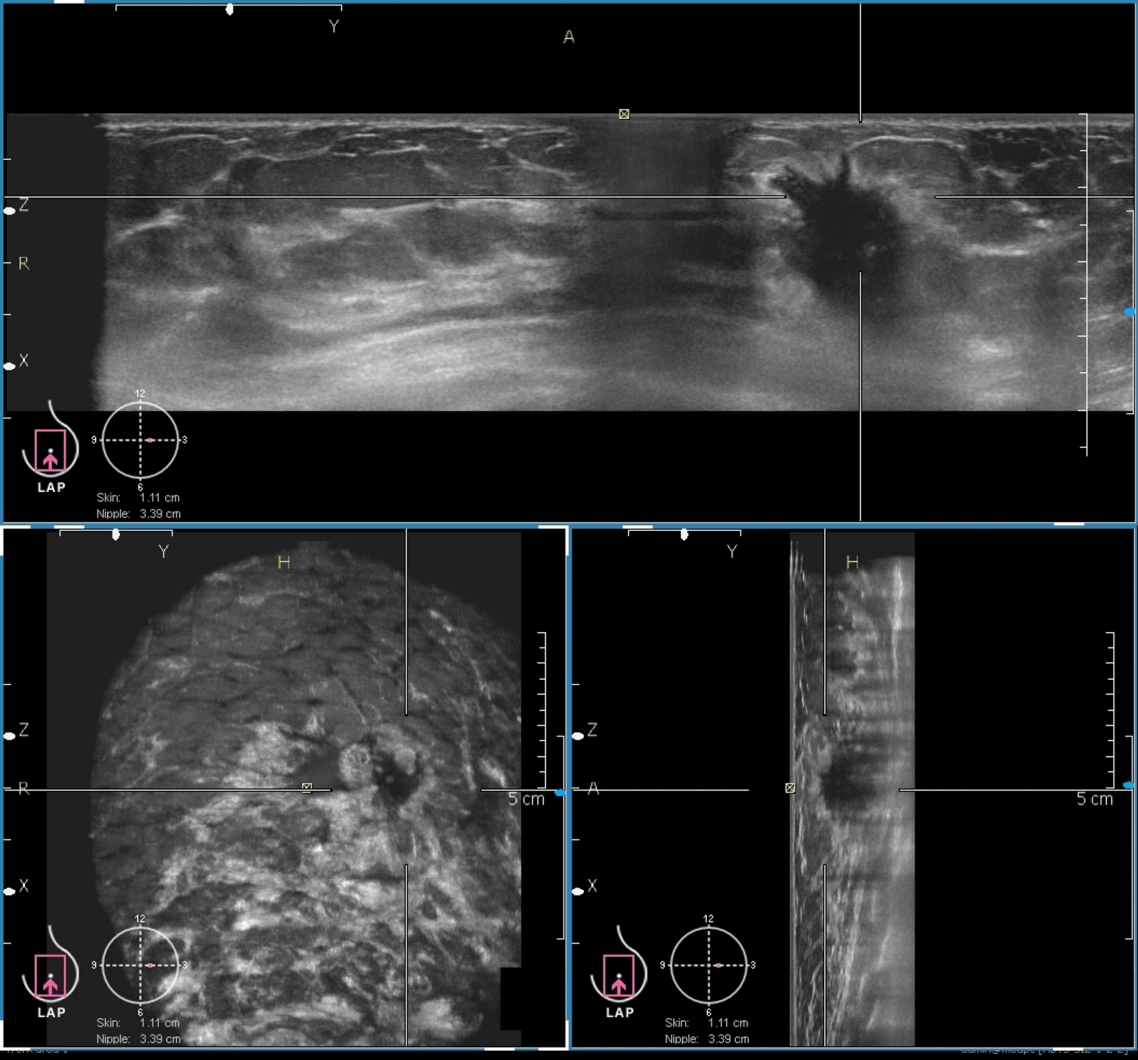
Comprehensive ABVS image of an irregular, hypoechoic, lesion displayed in three orthogonal planes The top panel shows the axial plane, the bottom right displays the sagittal plane, and the bottom left presents the coronal plane, which clearly demonstrates a spiculated lesion. A nipple marker is visible, aiding in assessing the lesion’s proximity to the nipple. The image also provides additional anatomical details, including lesion location within the breast and its relation to the skin and chest wall, allowing for evaluation of potential infiltration. (Histopathology: Infiltrating ductal carcinoma, Grade II). This is one of the study participants' images.

This case shows the comparable image quality of HHUS and ABVS. In addition, ABVS provides a three-plane view that shows the exact location of the lesion from the nipple, skin surface, and the chest wall. This information is of more value for surgical planning in malignant lesions. Figure 5 shows a malignant lesion depicted in the three-plane view.

Unique value of the retraction phenomenon in ABVS

**Figure 6:**
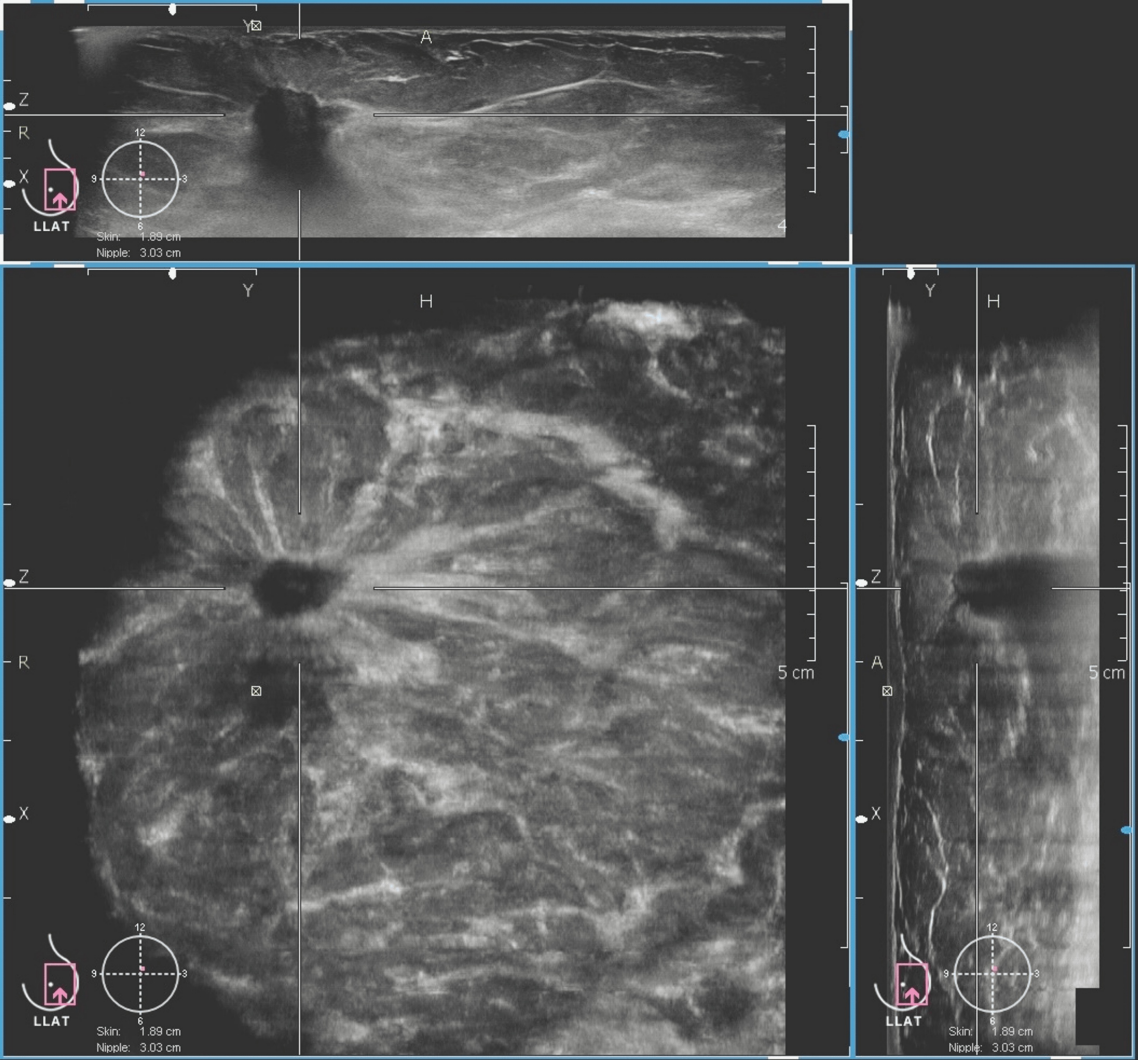
ABVS images demonstrating the retraction phenomenon in a patient included in this study The retraction phenomenon is characterized by converging bands of hyper- and hypo-echogenicity in coronal plane images of an automated breast volume scan (ABVS) in a case of infiltrating ductal carcinoma. The top panel demonstrates the axial plane; the bottom right displays the sagittal plane; and the bottom left shows the coronal plane, which is enlarged to clearly depict the retraction phenomenon, a feature suggestive of malignancy. A nipple marker is visible just inferior to the lesion, highlighting its anatomical relationship to the nipple and aiding in surgical planning.

A hallmark feature visible only in ABVS, the retraction phenomenon, proved to be 100% specific and had a PPV of 100% for malignancy in our study. However, its sensitivity was low (22%), suggesting that this feature is more common in advanced lesions. This aligns with reports by Chen et al. [18] and Wang et al. [21], who noted variable sensitivity but high specificity (Table 6). The retraction phenomenon, seen only in the coronal plane, is absent in HHUS and not currently included in the BI-RADS lexicon [19], yet it represents a powerful diagnostic clue in ABVS imaging (Figure 6).

**Figure 7:**
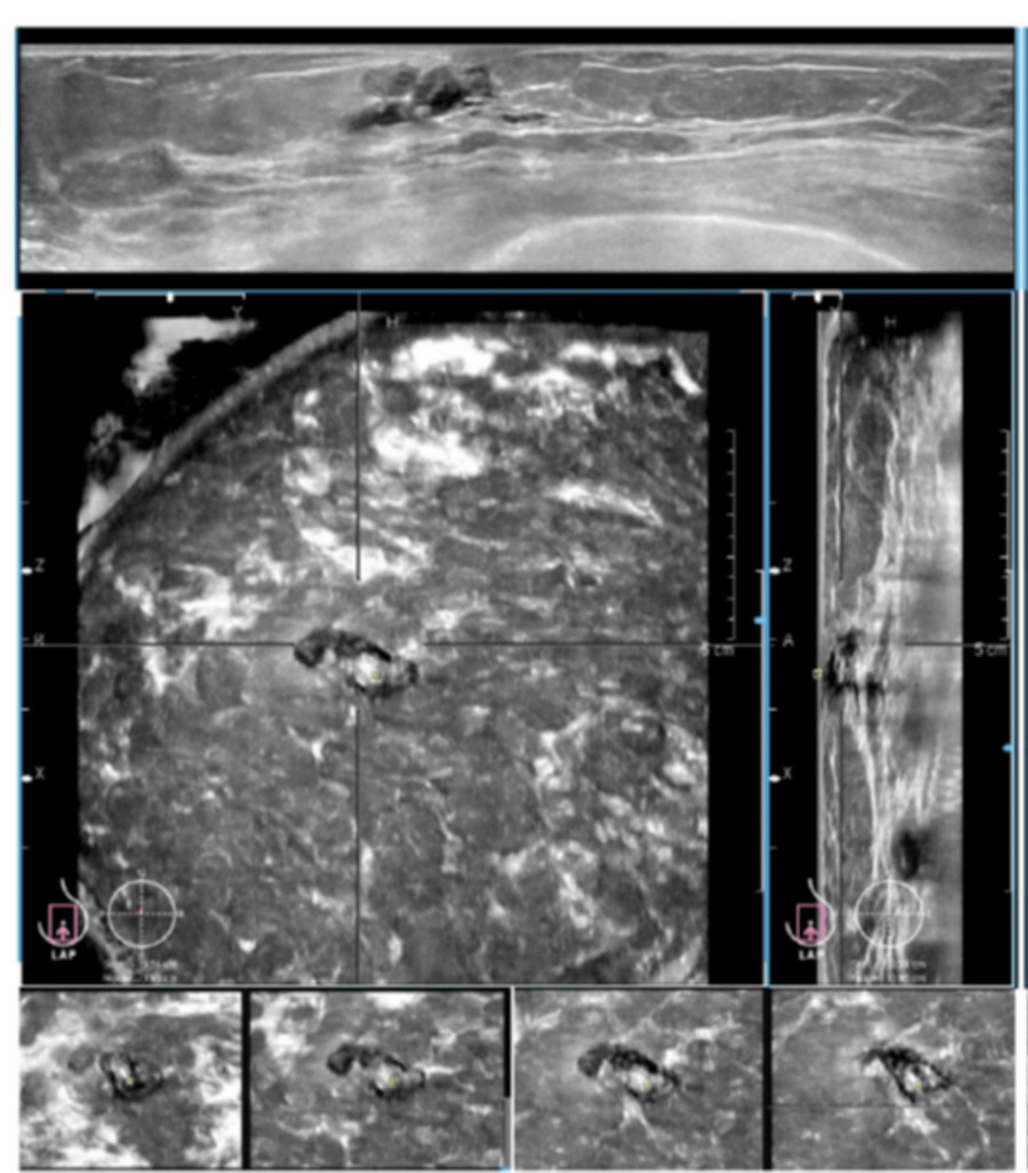
ABVS image of a patient included in this study Automated breast volume scanning (ABVS) image showing a dilated duct at the 10 o'clock position in the periareolar region reaching till the nipple with an intraluminal polypoidal lesion. The course of the duct is better visualised in the coronal plane images of ABVS. (Biopsy – intraductal papilloma).

Beyond malignancy prediction, the coronal plane adds diagnostic depth by showing the lesion’s relationship with surrounding structures, aiding in identifying intra-ductal pathology and architectural distortions more clearly than transverse or sagittal views (Figure 7).

Comparison between ABVS and HHUS

Our study found moderate to substantial agreement between ABVS and HHUS across multiple lesion descriptors. Agreement was substantial for shape, margin, cystic changes, and BI-RADS score, and moderate for orientation, calcification, acoustic shadowing, and architectural distortion. These findings are consistent with those of Zhang et al. [19] and Golatta et al. [22], who also reported strong inter-modality correlation. ABVS thus emerges as a viable and objective alternative to HHUS for breast lesion characterisation.

Inter-observer agreement in ABVS

One of the most compelling strengths of ABVS is its high interobserver reliability [22]. Our study found almost perfect agreement for shape, margin, orientation, posterior features, and BI-RADS assessment among observers. The echopattern showed substantial agreement. This consistency underscores the reproducibility and objectivity of ABVS, potentially reducing operator dependence and inter-reader variability often associated with HHUS.

Detection of additional lesions by ABVS

**Figure 8:**
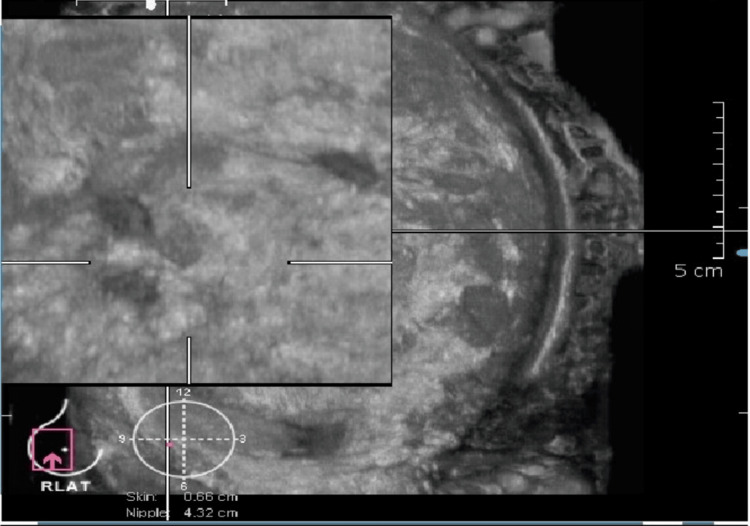
ABVS image of a 40-year-old patient A 40-year-old female presented with a lump in the right breast at the 9 o'clock position. A lesion was identified at 9 o'clock by HHUS. ABVS done for this patient showed two additional lesions in the magnified coronal plane image.

ABVS identified 21 additional lesions not seen on HHUS, 10 of which were classified as BI-RADS 4 or 5, with six confirmed malignancies. This finding directly impacted clinical decision-making and surgical planning. While Kim et al. observed a more mixed detection rate [23], our study suggests that ABVS may enhance lesion detection, particularly in dense breast tissue or multifocal disease (Figure 8).

Strength of the study

The study involves a direct comparison between ABVS and HHUS on the same set of patients, thus eliminating inter-patient variability. This enhances internal validity and ensures that the differences in the findings can be attributed to the imaging modality rather than patient-related factors. ABVS images were independently assessed by two experienced radiologists, enhancing inter-observer reliability and reducing subjective bias. The findings from both ABVS and HHUS were compared against histopathological analysis in every lesion, which served as the gold standard, ensuring strong validation of imaging results.

Study limitations

Despite promising results, our study has limitations. The sample size (n=101) restricts broad generalization, and larger prospective studies are needed to establish ABVS as a standard diagnostic tool or adjunct to mammography. Additionally, ABVS cannot evaluate axillary lymph nodes and lacks Doppler and elastography capabilities, which offer valuable information about vascularity and lesion stiffness [24-26]. The requirement for dedicated equipment and trained personnel also poses challenges for widespread implementation, especially in low-resource settings.

## Conclusions

Both ABVS and HHUS are effective tools for characterizing breast lesions, with comparable diagnostic accuracy. However, ABVS offers several distinct advantages: superior standardization, greater reproducibility, and the ability to evaluate the coronal plane, which enhances lesion characterization, particularly through features such as the retraction phenomenon, which are not visible on HHUS. Additionally, ABVS provides valuable spatial information, including lesion orientation and its distance from the chest wall, skin, and nipple, all within a single image, thereby aiding in preoperative surgical planning. Moreover, ABVS demonstrated higher sensitivity and was able to detect additional lesions missed by HHUS, including some that were histologically malignant.

While HHUS remains a widely available, cost-effective, and dynamic imaging modality, ABVS shows promise as a complementary tool, particularly in screening settings, follow-up evaluations, and scenarios where operator dependency and reproducibility are critical concerns. The high inter-observer agreement observed in ABVS interpretation further reinforces its consistency and diagnostic reliability. However, studies with larger patient populations are required to establish the feasibility of ABVS as an adjunct screening modality to mammography.
